# In-Memory Shellcode Runner Detection in Internet of Things (IoT) Networks: A Lightweight Behavioral and Semantic Analysis Framework

**DOI:** 10.3390/s25175425

**Published:** 2025-09-02

**Authors:** Jean Rosemond Dora, Ladislav Hluchý, Michal Staňo

**Affiliations:** Institute of Informatics, Slovak Academy of Sciences (IISAS), 84507 Bratislava, Slovakia; jeanrosemond.dora@savba.sk

**Keywords:** IoT security, in-memory malware, behavioral analysis, shellcode detection, system call monitoring, anomaly detection, endpoint security, lightweight agents, graph neural networks

## Abstract

The widespread expansion of Internet of Things devices has ushered in an era of unprecedented connectivity. However, it has simultaneously exposed these resource-constrained systems to novel and advanced cyber threats. Among the most impressive and complex attacks are those leveraging in-memory shellcode runners (malware), which perform malicious payloads directly in memory, circumventing conventional disk-based detection security mechanisms. This paper presents a comprehensive framework, both academic and technical, for detecting in-memory shellcode runners, particularly tailored to the unique characteristics of these networks. We analyze and review the limitations of existing security parameters in this area, highlight the different challenges posed by those constraints, and propose a multi-layered approach that combines entropy-based anomaly scoring, lightweight behavioral monitoring, and novel Graph Neural Network methods for System Call Semantic Graph Analysis. Our proposal focuses on runtime analysis of process memory, system call patterns (e.g., Syscall ID, Process ID, Hooking, Win32 application programming interface), and network behavior to identify the subtle indicators of compromise that portray in-memory attacks, even in the absence of conventional file-system artifacts. Through meticulous empirical evaluation against simulated and real-world Internet of Things attacks (red team engagements, penetration testing), we demonstrate the efficiency and a few challenges of our approach, providing a crucial step towards enhancing the security posture of these critical environments.

## 1. Introduction

The Internet of Things (IoT) revolutionizes several sectors, including smart homes, industries, and critical infrastructure (such as healthcare). Billions of interconnected devices, often with low computational power, battery life, and memory, form the backbone of this network. While these devices are incredibly beneficial to society, offering immense utilities and efficiency, their rapid proliferation and often inadequate security measures have created an open opportunity for cyber attackers. Conventional security mechanisms, designed for traditional IT environments, frequently fall short in protecting these IoT devices.

One of the most challenging and insidious threats in the IoT infrastructure is the in-memory shellcode runner. Unlike traditional malware, which relies on persistent files on disk, in-memory payloads execute directly within the memory space of a compromised process. This “fileless” nature makes it particularly difficult for conventional static analysis tools or signature-based antivirus (AV) to detect, as there are no disk artifacts to scan. However, after obtaining a reverse shell (remote access) on the target’s device, attackers may need to perform post-exploitation tasks (as is the case in most instances), which may necessitate using the PowerShell tool, even if they still perform a maximum of activities in memory. As a result, upon investigation, they may still get caught because the .NET framework and PowerShell tools leave artifacts. Attackers often use an in-memory payload as a second-stage shellcode, downloading and injecting scripts after an initial foothold. However, based on the criticality of the vulnerability, they may also use it as a first-stage attack (e.g., in a command injection (CI) attack). The ability to act solely in memory provides adversaries with a stealthy and transient niche, enabling device manipulation, data exfiltration, or even the formation of large-scale botnets. These specific constraints of IoT devices, “limited CPU, RAM, storage, and power,” further complicate the problem. Resource-intensive detection solutions standard in enterprise environments to thwart this type of attack are often complicated (and sometimes close to impossible).

Before diving into the detailed description, a shellcode is a small piece of machine code used as a payload (often with buffer data) in software exploits. Its fundamental purpose is to give an attacker control over a compromised target system, usually by launching a command shell (hence “shellcode”) or allowing remote access. In-memory shellcode refers to instances where this malicious code is injected and run directly in the process’s memory, avoiding the target device’s disk. This approach is a cornerstone of “living off the land” (LotL) attacks, where adversaries leverage benign system tools and memory to achieve their goals, blurring the lines between regular and malicious activity. Following this pathway, what is the main difference in shellcode runner detection between standard software and IoT devices?

Generally, the differences between detecting shellcode runners in standard software (i.e., Desktop/Server OS) and IoT devices are basically due to the unique features and constraints of IoT environments. Regular (normal or standard) software has a well-defined architecture and a rich set of system calls and application programming interfaces (APIs). Malware detection products can rely on a large volume of known signatures (disclosed hashes) and a wide range of behavioral analysis tools. The system often shows good characteristics, i.e., has ample power, computational resources, and memory to execute complex, resource-intensive detection agents. However, the IoT devices are highly diverse in terms of operating systems, hardware, and purpose. Unlike standard software, they do not exhibit those good characteristics, but rather have them in a limited form. This factor makes it impossible for IoT devices to run heavy-duty security agents. The API sets and system calls are often minimal and specific to the device’s operation, meaning conventional signature databases are less significant. Moreover, these devices function in a “hostile” environment, i.e., in disorder, and several of them lack a graphical user interface (GUI). Regular updates and patches are often missing, making them a high target for attacks.

The following challenges in designing a detection framework specifically for IoT are that they often lack “Heterogeneity, Ground Truth Data”, and “Scalability”. The first one can be explained as follows: due to the vast variety of these devices, it is challenging to design a one-size-fits-all solution. That is, a single model tested on one type of device may not be compatible or effective for another. The second missing point, “Ground Truth Data”, refers to the fact that, due to their diverse features, it is challenging to build a comprehensive dataset for anomaly detection that accurately represents the variety of IoT device behavior. For the “scalability”, this feature requires that the framework must be able to scale to monitor hundreds of millions of devices in a very cost-effective manner, which IoT devices do not have.

Artificial intelligence (AI), combined with ML, can be very beneficial in malware detection and classification. This categorization is the subject of ML, and classifiers such as the Logistic Regression, Naïve Bayes Classifier, and K-Nearest Neighbors [1]. Security engineers utilized Convolutional Neural Networks (CNNs), Hidden Markov Models, Random Forests, natural language processing methods (NLPs), and Support Vector Machines (SVMs) to detect malware, aiming to achieve improved performance. However, the traditional and conceptual use of ML algorithms is trivial in identifying this type of malware. As such, defenders are called to adapt to the evolving techniques used by attackers to increase the detection rate of security solutions; however, in a reverse manner. This “arms race” is enhancing the robustness of AI-based malware detection and making it more appealing. For more information, please refer to [2,3,4]. NLP techniques are used to visualize the internal structure of the malware by extracting keywords from the textual representation and hooking the semantics of the text. That is to say, NLP identifies the evidence of malicious content in the network traffic. It is an interesting approach for researchers in the domain of malware detection. Similarly, NLP algorithms such as Term Frequency-Inverse Document Frequency (TF-IDF) matrices, FastText, Bag-of-Words (BOW), and Word2vec may also be used to identify the structures of malware data. NLP methods extend their services with advanced deep learning (DL) models [5,6] for textual data processing and classification tasks, utilizing transformer models that possess the capability to process large and complex data. (Please refer to [7,8,9,10] for more detail about the technologies aforementioned.)

Despite the benefits of ML and deep learning models, challenges have arisen in malware detection and classification. Imbalanced data and high-dimensional malware data can seriously impact the performance of these tools. The structure and nature of advanced malware represent a key challenge for them to provide accurate detection results. However, the dataset plays an important role in the success of the DL models. The availability of large datasets represents a key factor in the success of NLP. Malware injection can affect any operating system (Windows, Linux, macOS, Android, etc.). Nonetheless, some of them offer higher native security standards than others; also, their patched versions play a significant role in the security base. For example, Android applications (Apps) can be harmed by network connectivity issues. Since these apps typically operate over the network, and malicious content also travels through network traffic, they can be easily compromised. From this perspective, it is of high interest that researchers are also concerned with analyzing network traffic to identify rarities, artifacts on the system, and further to detect the presence of malware. (Please see [11,12,13,14] for more information.)

Since malware can be launched against any device within a network, regardless of the target operating system (OS) in use, it is crucial to monitor and apply security solutions to every component that performs egress and ingress traffic, including all IoT devices. Often, these devices are overlooked in favor of security patches, which can have a significant impact on the system. However, malware should be programmed to target a specific OS. A Windows malware will generally not work on a Linux device, and vice versa.

### Contributions

Armed with all the benefits that ML, DL, AI, and security solutions provide in detecting malware and mitigating its propagation, this paper addresses this critical gap by proposing a novel, lightweight, and compelling framework for detecting in-memory shellcode runners in IoT networks. We also underscore behavioral anomaly detection and ML tailored for the unique, typical features of these devices. Our descriptive proposed framework is outlined as follows:

- Implementing a lightweight, resource-efficient on-device agent tailored for IoT limitations, focusing on critical low-level memory and process telemetry.
- Introducing adaptive entropy-based rarity scoring to detect suspicious memory regions without static thresholds accurately.
- Proposing a hybrid edge-fog-cloud scheme that intelligently offloads sophisticated computations to more advanced backend systems, enabling complex analysis without burdening edge devices.
- Lastly, pioneering the use of the Graph Neural Network (GNN) to analyze and model the semantic and causal relationships within system call functions (events), providing a comprehensive understanding of process behavior beyond traditional sequential patterns.

The rest of the paper is structured as follows: In Section 2, we provide a background on the in-memory shellcode runner in IoT networks. We evaluate our malware detection in comparison to other works in closely related areas. Section 3 embraces our proposed multi-layered detection framework for IoT devices. It details the importance, implementation, and usage of the “Lightweight Behavioral and Semantic Analysis”. Section 4 focuses on the experimental setup of the laboratory, data aggregation, performance metrics, and other related topics. In Section 5, we highlight the novelty and innovations of this research. Section 6 provides a short discussion and focuses on our future work. Section 7 concludes the paper.

## 2. Background and Related Work

The escalating refinement of cyber threats, especially those leveraging in-memory execution, raises significant security questions about protecting the rapidly expanding IoT network. Conventional security paradigms, often reliant on disk-based rarities, are increasingly ineffective against these types of attacks. This section elaborates on the techniques that attackers employ to perform in-memory shellcode attacks. We review existing research pertinent to malware detection, in-memory attack methods, and IoT security, underscoring the constraints that our proposed framework aims to address.

### 2.1. Techniques to Execute In-Memory Attacks

Common techniques for injecting and executing in-memory shellcode include:

- Process Injection—Injecting code into another running application process’s memory space (e.g., using ptrace on Linux, VirtualAllocEx and WriteProcessMemory on Windows OS, IoT) [15].
- Reflective dynamic-link library (DLL) Loading—Loading a DLL directly into memory without it being present on disk.
- Memory Corruption Exploits—Vulnerabilities like buffer overflows can be abused to overwrite return addresses, redirecting execution flow to adversary-controlled shellcode injected into memory.
- Just-In-Time (JIT) Spraying: Injects scripts into a browser’s Just-In-Time (JIT) compiler, causing it to compile and execute damaging native code. While less common on limited IoT devices, it is a theoretical pathway for devices with rich web runtimes.

Note that the main motivation for attackers using in-memory shellcode techniques is evasion and stealth. By sidestepping disk writes, they circumvent file-based detection products, forensic tools, and conventional logging mechanisms, which pose significant challenges for incident response tools.

### 2.2. In-Memory Malware, Attack Vectors and Fileless Attack Detection

Research into detecting in-memory malware has focused on enterprise computing systems, addressing techniques such as ML, memory forensics, and behavioral analysis. Early approaches focused on memory forensics, where system memory dumps from devices are analyzed offline to detect malicious code, injected modules, or data structures. While tools like Volatility have been influential in this domain, enabling the extraction of network connections, process lists, and loaded DLLs, the resource-intensive nature of memory dumping and analysis makes these techniques inadequate for real-time, on-device detection in limited IoT networks [16]. Authors of [17] proposed taking snapshots of process virtual memory and feeding them to a payload detector for investigation, aiming for low overhead. However, the direct functionality of minimal IoT devices needs further analysis.

Heuristic analysis has emerged as a promising alternative for malware detection, focusing on the dynamic activities (functionalities) of the invoked processes. That includes monitoring for suspicious system calls, unusual memory access patterns, and API hooking. Other authors have investigated the detection of code injection by monitoring for modifications in memory page permissions, such as pages becoming simultaneously writable and executable. Entropy analysis, which quantifies the randomness of a memory location, has also been widely addressed, as encrypted or compressed payloads often display high entropy values close to 8 bits per byte. A recent work [18] explores intelligent intrusion detection systems (IDSs) that utilize artificial neural networks, addressing the challenge of distinguishing the structure of the payload from legitimate network traffic due to its obfuscated nature.

More recently, the ML tool has been applied to analyze behavioral and heuristic characteristics for detecting fileless malware. Gond et al. (2025) [19] provided a comprehensive overview of system calls for malware detection, covering both static and dynamic analysis, as well as the usability of ML techniques for pattern or structure analysis. Similarly, the [20] paper highlights the significant surge in fileless malware and LotL methods, emphasizing the need for behavior-based detection techniques that extend beyond signature-based software. Approaches range from typical ML algorithms trained on sequences of system calls to DL models for more complicated design recognition [21]. Authors of this paper [22] proposed a compelling technique for improving malware detection in PDF files by addressing active learning, which, although not directly related to shellcode, is necessary for model patches in behavioral cybersecurity systems relevant to fileless threats. Notwithstanding, adapting these models for resource-constrained IoT devices often requires meticulous feature selection and eminently optimized architectures.

### 2.3. Limitations, Security Challenges of Malware Detection in IoT Networks

The unique features of IoT devices, i.e., their resource constraints such as limited CPU, battery, and memory, as well as the heterogeneity of hardware and often insecure update mechanisms, present significant hurdles for implementing robust security mechanisms [23]. Conventional cybersecurity solutions are often incompatible with these types of network systems. Research in IoT malware detection has investigated several different angles:

- Signature-based detection: A common technique, however, suffers from the same constraints as in traditional IT, failing against zero-day attacks or polymorphic threats, especially in-memory shellcode (variants). This security solution is based solely on comparing malicious hashes (of offsets in binaries) with those of a target file being scanned.
- Traditional Heuristics: Often too resource-intensive for continuous device monitoring. That may also be prone to false positive alerts.
- Full Memory Forensics: This approach necessitates significant RAM, CPU, and storage for memory analysis, rendering it incompatible for most IoT devices.
- Network-based intrusion detection systems (NIDS): This security layer monitors traffic for known attack patterns or anomalies in IoT networks (for example, Message Queuing Telemetry Transport (MQTT), Constrained Application Protocol (CoAP)) [24]. While valuable for discovering command and control (C2) communication or DDoS activities, NIDS cannot detect in-memory shellcode before network-level indicators manifest.
- Host-based intrusion detection systems (HIDSs): This measure usually focuses on process activity, lightweight monitoring of system logs, or basic network connections. Some other proposals used system call monitoring, analyzing sequences of calls to implant a baseline of normal behavior and identify deviations. However, as we previously mentioned, these functionalities often struggle with the complexity and stealth of in-memory shellcode, which can successfully mimic genuine activities (e.g., attackers can use the Hollowing process [25] for this purpose).

#### Challenges of IoT Security

IoT networks face numerous security challenges. Some of them are listed as follows:

- Resource Constraints: Limited CPU, battery, storage, and memory capacity make conventional heavy-duty security solutions inadequate.
- Heterogeneity: Various hardware architectures, such as ARM, MIPS, RISC-V, OS (Linux variants, RTOS, custom firmware), and communication protocols, complicate unified security layers.
- Obsoleteness and Lack of Standardization: Fragmented improvement and deployment models lead to inconsistent and obsolete security practices that are in use. That is, many IoT devices lack strong, or even any, over-the-air (OTA) update features, leaving them vulnerable to common exploits.
- Headless Operation: The absence of a user interface restricts direct user interaction for security notifications or configurations.
- Remote Management: Devices are often deployed in inaccessible or remote regions, making physical access for incident response challenging.

Our experiment for this paper builds upon these foundations, adapting and innovating methods to especially address the problem of in-memory shellcode detection within the unique limitations of IoT devices.

## 3. Proposed Framework: Lightweight Behavioral and Semantic Analysis

**Figure 1:**
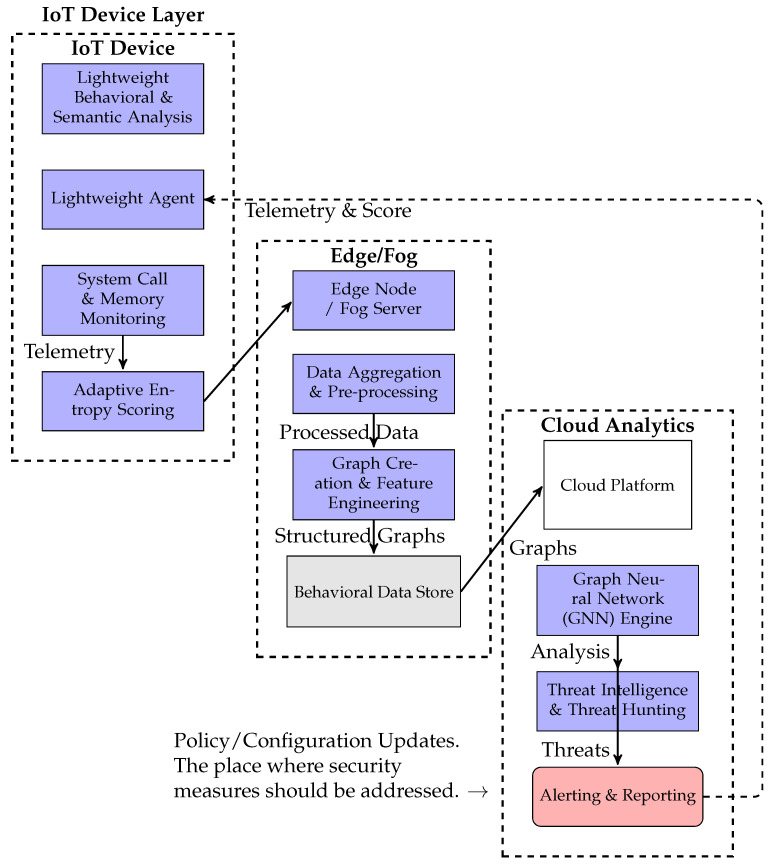
Proposed multi-layered detection framework for in-memory shellcode in IoT networks.

Our proposed framework, illustrated conceptually in Figure 1, encompasses several interconnected modules designed to function efficiently on resource-constrained IoT devices. It is subdivided into three main blocks, which are “IoT Device Layer, Edges, Cloud analytics”. The first block depicts the phase where the system calls, and Win32 API functions can be invoked. The performance of the memory and the entropy scoring can also be addressed. The second block deals with the construction of the graph by phases. The final segment illustrates threat intelligence analysis and hunting, as well as countermeasures.

### 3.1. Lightweight On-Device Agent (Edge Layer)

The bottom line of our on-device detection lies in an eminently optimized, low-footprint agent built for various types of IoT architectures (for instance, MIPS, ARM). This agent emphasizes the collection of specific and valuable telemetry indicative of in-memory attacks.

#### Core Functions

- Process Memory Monitoring: The agent periodically scans specific memory locations (regions) and observes changes in memory (e.g., type of permissions).
  – Writable-Executable ($W^{X}$) Protection–Detection: Monitors for memory instances that are simultaneously writable and executable. Benign code usually resides in executable-only (EO) protection, while data resides in writable-only (WO) protection. A protection becoming $W^{X}$ (e.g., via mprotect on Linux) is a strong indicative factor of potential shellcode injection.
  – Entropy-based Anomaly Scoring (EAS): For newly apportioned or permission-altered executable memory regions, the agent calculates the value of the Shannon Entropy. Malicious shellcode often exhibits high Entropy (randomness), as previously mentioned Section 2.2, due to its compressed nature or its random behavior, unlike ordinary program code. We can calculate the Shannon entropy function for a sequence of bytes SB, which is defined as follows:
    (1) $$H\left(SB\right)=-\sum_{i=1}^{n}P\left({\left(sb\right)}_{i}\right)\log_{2}P\left({\left(sb\right)}_{i}\right)$$
    where *n* is the number of probable byte values (256), and $P({\left(sb\right)}_{i})$ is the likelihood of byte ${\left(sb\right)}_{i}$ occurring in the sequence. A higher value expresses greater randomness.
    The Anomaly Score, $S_{entropy}$, is then derived from the computed entropy:
    (2) $$S_{entropy}\left(H\left(SB\right)\right)=\left\{\begin{array}{cc} \left(H\left(SB\right)-T_{low}\right)/\left(T_{high}-T_{low}\right) & \mathrm{if}\;T_{low}<H\left(SB\right)<T_{high}\\ 1 & \mathrm{if}\;H\left(SB\right)\geq T_{high}\\ 0 & \mathrm{if}\;H\left(SB\right)\leq T_{low} \end{array}\right.$$
    Here, in this case, $T_{low}$ and $T_{high}$ are empirically defined entropy thresholds. Values between that range are scaled; values below $T_{low}$ score 0 (legitimate). Likewise, values above $T_{high}$ score 1 are highly suspicious. Novelty: We propose adaptive thresholds for EAS based on device-specific baseline entropy profiles. That is, instead of static $T_{low}/T_{high}$, these can be mean μ and standard deviation σ derived from the normal behavior of devices, flagging regions where H(SB)>μ+kσ.
- System Call Hooking: The agent hooks critical system calls (e.g., read, write, mmap, socket, mprotect, execve, ptrace, connect, kill). It logs Syscall ID, Thread ID (TID), Process ID (PID), Timestamp, and Return Status, and chooses key arguments, for example, destination IP/port for connect, and memory address/permissions for mprotect.
- Control Flow Monitoring (Lightweight): For devices without hardware-assisted control flow integrity (CFI), the agent can track for unexpected control flow transfers to non-code memory locations within a process using simplified instruction monitoring or analyzing stack behavior.

For the “HandleMprotectEvent” procedure, we utilize function calls, process identifiers (i.e., PID, addresses, and length) to calculate the telemetry type. Parameters such as PID, TID, addr, len, and rights, from the “HandleMmapEvent” procedure, were handled and mapped to evaluate the security policy. For the “BackgroundMemoryScanThread” procedure, we performed a background scan on threads in order to estimate the Shannon entropy. For the “EnqueueTelemetrydata” procedure, we invoked the sequences to facilitate working with buffer generation and encryption techniques. Please see the following Algorithm 1.
**Algorithm 1** Lightweight On-Device Agent Pseudocode1:**procedure** InitializeAgent2:    HookSyscall(“mprotect”, HandleMprotectEvent)3:    HookSyscall(“mmap”, HandleMmapEvent)4:    HookSyscall(“execve”, HandleExecveEvent) ▹ *//... Other critical syscalls can also be added here. Please see the "System Call Hooking" from the Section 3.1.*5:    StartBackgroundMemoryScanThread()6:**end procedure**7:**procedure** HandleMprotectEvent(PID, TID, addr, len, rights)8:    **if** rights includes PROT_EXEC and PROT_WRITE **then**9:        EnqueueTelemetry({Type: “$W^{X}$_PROTECTION”, PID: PID, Addr: addr, Len: len, Perms: rights, Timestamp: Now()})10:        *current_entropy* = CalculateEntropy(PID, addr, len)11:        **if** *current_entropy* > LocalEntropyThreshold **then**12:           EnqueueTelemetry({Type: “HIGH_ENTROPY_EXEC”, PID: PID, Addr: addr, Len: len, Entropy: *current_entropy*, Timestamp: Now()})13:        **end if**14:    **end if**15:**end procedure**16:**procedure** HandleMmapEvent(PID, TID, addr, len, rights)17:    **if** rights includes PROT_EXEC and (rights includes PROT_WRITE or PROT_READ) **then**18:        EnqueueTelemetry({Type: “MMAP_SUSPICIOUS”, PID: PID, Addr: addr, Len: len, Perms: rights, Timestamp: Now()})19:        *current_entropy* = CalculateEntropy(PID, addr, len)20:        **if** *current_entropy* > LocalEntropyThreshold **then**21:           EnqueueTelemetry({Type: “HIGH_ENTROPY_EXEC”, PID: PID, Addr: addr, Len: len, Entropy: *current_entropy*, Timestamp: Now()})22:        **end if**23:    **end if**24:**end procedure**25:**procedure** BackgroundMemoryScanThread26:    **loop**27:        **for** each running PID **do**28:           QueryProcessMemoryMaps(PID)29:           **for** each memory region in map **do**30:               **if** region/location is Executable AND was previously WO-RO **then**31:                   *current_entropy* = CalculateEntropy(region.data)32:                   **if** *current_entropy* > LocalEntropyThreshold **then**33:                       EnqueueTelemetry({Type: “HIGH_ENTROPY_EXEC”, PID: PID, Addr: region.start, Len: region.size, Entropy: *current_entropy*, Timestamp: Now()})34:                   **end if**35:               **end if**36:           **end for**37:        **end for**38:        Sleep(Scan_Interval_Seconds)39:    **end loop**40:**end procedure**41:**procedure** EnqueueTelemetry(data)42:    Add *data* to a small, circular buffer *TelemetryBuffer*.▹ *(//Staged payloads generated from msfvenom, for example. Please see [26] page 1225, and [27] for more information.*43:    **if** *TelemetryBuffer* size reaches threshold OR timeout **then**44:        *compressed_encrypted_data* = CompressAndEncrypt(*TelemetryBuffer.content*)45:        SendTelemetryToFogOrCloud(*compressed_encrypted_data*)46:        Clear *TelemetryBuffer*47:    **end if**48:**end procedure**

### 3.2. Fog Layer/Network Gateway (Pre-Processing and Aggregation)

For larger IoT deployments, an intermediary fog layer (e.g., a gateway device) can aggregate telemetry from several restricted edge devices, execute initial filtering, and perform lightweight pre-processing. It decreases network bandwidth to the cloud and provides quicker regional response capabilities. Note that “Fog networking” is a type of decentralized architecture that brings cloud computing capabilities to the network’s edge, increasing efficiency, improving data processing capabilities, and minimizing latency.

- Telemetry Aggregation (assemblage): Consolidates similar events from different devices.
- Initial Feature Engineering: May merge related events, for instance, a sequence of mmap and mprotect calls) into more advanced features.
- Lightweight Machine Learning Inference: Can host small, predefined ML models (e.g., a Logistic Regression classifier or a Decision Tree) to filter out frequent false positives or detect obvious suspicious patterns before transferring them to the cloud.
- Encrypted Communication: Ensures the safety and security of sensitive data in transit to the central (e.g., cloud) analytics platform.

### 3.3. Cloud-Based Semantic Analysis Engine (Backend Layer)

This powerful layer performs in-depth analysis on aggregated telemetry using advanced ML models and global threat intelligence.

#### Key Innovations

- System Call Semantic Graph Analysis (SCSGA): This represents a core novel element. Instead of just invoking and evaluating sequences of system calls, we construct a “dynamic graph depiction of process behavior”.
  – Graph Structure: Each system call event, such as PID, Arguments, Syscall ID, Timestamp, becomes a node. Edges represent connections. Some of them are listed as follows:
    * Temporal Edge: This edge connects sequential “syscalls” within the same thread.
    * Memory Access Edge: This one links a syscall (e.g., read, write, mmap, mprotect) to the specified memory region it acts upon, thus modeled as a separate node.
    * Process/File Edge: This file edge links syscalls related to process creation, for example, “execve, fork” or file access such as “read, write” and “open”.
    * Inter-process Communication, also known as IPC Edge: It links syscalls related to sockets, pipes, and shared memory between processes.
  – Semantic Graph Representation: The graph nodes are enhanced with characteristics derived from the syscall arguments, for instance, memory protection flags, file descriptor, entropy score from edge layer, and network addresses.
- Graph Neural Network (GNN) for Anomaly (rarity) Detection: We address a customized GNN (Graph Convolutional Network (GCN), for example) or Graph Attention Network (GAT)) to learn robust, nonlinear patterns, structures within these behavioral graphs.
  Note: The decision of using the two-layer GCN instead of more complex models like temporal GNNs or attention-based graph models (e.g., StrGNN) is driven by a trade-off between model intricacy, computational effectiveness, and detection performance in a limited IoT environment. Several factors were considered for this decision, detailed as follows:
  – Reduced Computational Overhead: These complicated models (e.g., StrGNN) require significantly more computational resources for testing, inference, and training. In an IoT environment, where devices may have constrained memory, CPU, and battery life, a lightweight model is vital. A simple two-layer GCN is computationally less demanding, making it more feasible to run in a centralized cloud backend or on edge devices without causing excessive CPU usage or latency. This directly aligns to minimize CPU overhead.
  – Addressing the Specific Problem: The core issue is identifying rarities based on a device’s behavioral graph, for example, system calls and memory events. While temporal and attention-based models are powerful at capturing complex sequential dependencies, a static GCN model is often sufficient for this task. It learns to analyze and classify a snapshot of a device’s behavior as either normal or suspicious by leveraging the rapport between nodes (e.g., files, processes) and their characteristics within a single graph. The static (unchanging) nature of the GCN is a deliberate design choice that assumes the relevant malicious patterns can be detected from a single behavioral graph rather than from a long series of such graphs.
  – Directness and Interpretability: A simpler GCN model is less complicated to design. It facilitates smoother training and debugging. In the context of a security system, where false positive alerts can be critical, understanding why a model flagged an anomaly is significantly beneficial.
  While more advanced models may provide slight improvements in accuracy, they often come at a high cost in terms of resource consumption and performance, which can be a prohibitive factor in real-world IoT deployments. The decision of using a simple GCN indicates a prioritization of practicality and efficiency (adaptability) over maximizing every last allotment point of detection rate.
  The following example is an update rule formulated for a single layer of a Graph Convolutional Network (GCN):
  (3) $$M^{\left(l+1\right)}=\sigma \left({\tilde{D}}^{-1/2}\tilde{A}{\tilde{D}}^{-1/2}M^{\left(l\right)}W^{\left(l\right)}\right)$$
  where
  – $M^{\left(l\right)}$: Input feature matrix for a layer, denoted as *l*. Each row corresponds to a node, i.e., a syscall event, and columns are its characteristics (features).
  – $M^{\left(l+1\right)}$: Defines the output feature matrix for the layer l+1.
  – $\tilde{A}=A+I$: Adjacency matrix (square matrix) of the graph with supplementary self-loops (I) represents the identity matrix, allowing nodes to collect (aggregate) their features.
  – $\tilde{D}$: Diagonal degree matrix of square matrix $\tilde{A}$, where ${\tilde{D}}_{ii}=\sum_{j}{\tilde{A}}_{ij}$. The ${\tilde{D}}^{-1/2}\tilde{A}{\tilde{D}}^{-1/2}$ concept normalizes the square matrix, preventing features from disappearing during the gathering.
  – $W^{\left(l\right)}$: This rule is a learnable weight matrix for the layer *l*. It transforms the collected features.
  – σ: This is a “Nonlinear activation function”. It introduces non-linearity to learn convoluted patterns.
  This equation describes how node features are collected from their neighbors and transformed through a learnable linear alteration, followed by a non-linearity. The final layer of the GNN assigns an anomaly score to the complete graph or specific subgraphs, indicating the likelihood of malicious in-memory shellcode activity. This layer allows defenders to detect sophisticated, multi-stage attacks that might individually appear legitimate but form a malicious behavioral pattern when observed holistically across the system call graph.
  Novelty: For the “in-memory shellcode” detection, we are applying GNNs to model the “semantic relationships” and “causal dependencies” of system calls within a process’s lifecycle. That goes beyond simple N-gram examination by capturing more important contextual data and nonlinear cooperation, which is improved for sparse IoT telemetry.
- Integration with Global Threat Intelligence: This step involves continuous updates/patches and fine-tunes the GNN models based on new shellcode variants and C2 infrastructures from global threat feeds.
- Forensic Analysis and Visualization: Provides tools for cybersecurity analysts and professionals to visualize the identified anomalous graphs, drill down into well-defined system call events, and start incident response procedures to isolate compromised devices, for example.

We develop a graph neural network to detect anomalies and misbehavior of an IoT device caused by the presence of malware (shellcode runner) and potential artifacts. Based on the environments (such as the seeds where devices are constantly running or the cloud backend), we can generate the Algorithm 2 according to the infrastructure, respectively.
**Algorithm 2** GNN-based Anomaly Detection—Cloud Backend1:**procedure** TrainGNNModel(TrainingGraphsLegitimate, TrainingGraphsMalicious)2:    Initialize GNN design ▹ *//2-layer graph convolutional network with defined hidden dimensions.*3:    **for** each *graph* in (*TrainingGraphsLegitimate* ∪ *TrainingGraphsMalicious*) **do**4:        Convert *graph* into Node_Feature_Matrix (**X**) and Adjacency_Matrix (**A**)5:        *Predicted_Score* = GNN(**X**, **A**, LearnedWeights)6:        *Loss* = CalculateLoss(*Predicted_Score*, *graph.TrueLabel*)7:        Update LearnedWeights using Backpropagation8:    **end for**9:    **return** Trained_GNN_Model, Optimized_Detection_Threshold10:**end procedure**▹*//Detecting anomalies, rarities, invoke system calls.*11:**procedure** DetectAnomaly(NewTelemetryData)12:    Convert *NewTelemetryData* (syscalls, memory events) into Behavioral_Graph (*G_new*)13:    Convert *G_new* into Node_Feature_Matrix ($X_{new}$) and Adjacency_Matrix ($A_{new}$)14:    *Anomaly_Score* = Trained_GNN_Model ($X_{new}$, $A_{new}$, LearnedWeights)15:    **if** *Anomaly_Score* > Optimized_Detection_Threshold **then**16:        **return** “Malicious In-Memory Shellcode discovered”, *Anomaly_Score*, *G_new*17:    **else**18:        **return** “Legitimate Activity”, *Anomaly_Score*19:    **end if**20:**end procedure**

### 3.4. Methodology for Deriving the Threshold

The most common technique for determining an optimal detection threshold is Receiver Operating Characteristic (ROC-based) tuning. The ROC curve outlines the detection rate, i.e., the true positive rate, against the false positive rate at various threshold settings (see Section 4.3). Each point on the curve represents a different potential threshold. The optimal threshold is selected from this curve based on a specific objective, such as:

- Maximizing a metric: Locating the threshold that yields the highest F1-score, which balances precision and true positive rate (recall).
- Balancing trade-offs: Choosing a point that achieves an acceptable trade-off between the detection rate (recall, TPR) and the rate of false alerts, which is paramount in a security system to avoid unnecessary alerts.
- Tuning to a particular false positive rate (FPR): Establishing the threshold to ensure the system does not exceed a predefined false positive rate.

The threshold selection strategy suggests that the proposed framework assigns a numerical score to identified rarities (anomalies). A decision is then made based on this score. If the score exceeds a certain pre-defined or dynamically determined threshold, the “Response Module” will be triggered to take action, such as process termination, immediate blocking, or isolation.

### 3.5. Adversarial Testing

At the beginning of the paper, we mention that the proposed framework’s effectiveness is demonstrated through a “meticulous empirical evaluation against simulated and real-world Internet of Things attacks (red team engagements, penetration testing).” That indicates that we cannot assume anything without testing the target devices. This phase is a core part of the validation process, which reveals any presence of vulnerabilities and their potential exploitation without malicious intent. These types of assessments employ specific and practical methods of adversarial testing that simulate real-world attacks to evaluate the security posture of IoT networks rigorously.

## 4. Experimental Setup and Evaluation

To validate our proposal framework, a comprehensive experimental setup was conducted, involving a variety of IoT devices and testbeds, and a penetration test was performed against them.

### 4.1. Testbed Environment

- IoT Devices: A heterogeneous aggregation of current IoT devices such as ESP32 (Xtensa), from manufacturer: Espressif Systems, City: Shanghai, Country: China. The raspberry Pi 4 (ARM) from manufacturer: Sony UK Technology Centre (under contract with the Raspberry Pi Foundation), City: Pencoed, Country: Wales, United Kingdom. An industrial controller (MIPS-based Linux). Those accessories usually ran custom Linux distributions, such as Buildroot/Yocto or FreeRTOS.
- Network Setup: We implement, configure, and use a restrained hands-on lab IoT network segment with isolated VLANs to be able to connect to a cloud backend for telemetry submission.
- Attack Toolkit: Kali Linux OS was used as the attacking machine. Shellcode generation tools, such as Metasploit Framework’s msfvenom and custom assembly code using objdump, were used. This last listed program is a command-line tool that exhibits various information about object files on Unix-like OS. We also used the nasm, a Netwide Assembler (NASM) and disassembler for the Intel ×86 architecture.
  Various abusive attempts were made to exploit vulnerabilities in those IoT devices. For example, Process injection through MQTT brokers, buffer overflows in obsolete web servers, and command injection were some of them. Different shellcode payloads (e.g., reverse shells, data exfiltration scripts, lightweight DDoS botnet agents, simple cryptominers) were launched, according to the target device’s architecture.
- Legitimate Workloads: We also consider performing benign device activity during our experiments to implement accurate baselines and evaluate false positive rates; for example, actuator control commands, secure shell (SSH) administration sessions, periodic sensor data logging (MQTT/HTTP), and routine firmware update checks.

### 4.2. Data Collection

Telemetry was aggregated by the on-device agent ($W^{X}$ warnings, estimated entropy scores, crucial syscall sequences with important arguments) for both legitimate and malicious activities over several days. The ground truth of those activities was meticulously recorded for all executed attempts. Furthermore, this dataset was used to test the GNN models at the cloud backend.

### 4.3. Performance Metrics

The system’s performance was rigorously calculated using standard cybersecurity metrics:

- Detection Rate (True Positive Ratio—TPR): $TPR=\frac{TP}{TP+FN}$. Value FN defines false negative alerts.
- False Positive Ratio (FPR): $FPR=\frac{FP}{FP+TN}$ (minimizing FPR is recommended for IoT devices to avoid disrupting vital operations).
- Precision: The precision, i.e., the exactness of detection, can be evaluated by the true positive alerts divided by the sum of true positive and false positive. $\frac{TP}{TP+FP}$
- Recall (equivalent to TPR): $\frac{TP}{TP+FN}$
- F1-Score: $2\times \frac{Precision\times Recall}{Precision+Recall}$ Generally F-score is a measure of predictive performance. It is computed from the precision and recall of the test, where precision is the numerical value of true positive alerts divided by the total number of cases predicted to be positive, including those not detected correctly. In brief, it is the harmonic mean of precision and recall.
- Latency Detection: Time taken from shellcode execution to be discovered at the cloud backend, which maintains the countermeasures.
- Resource Overhead on Device: This is closely related to the device power (CPU), network bandwidth, etc.
  – CPU Usage: The CPU work is evaluated as a percentage. It increases during various agent operations and on different devices.
  – Network Bandwidth: Average data transmitted by the agent, usually measured in megabytes per hour (MB/hour).
  – Memory Footprint: This is usually referred to Resident Set Size (RSS). It is the portion of memory (measured in KB) held by a process in the core memory (RAM) of the agent process.

### 4.4. Comparative Analysis

The proposed framework’s performance was compared against various security techniques, from two (2) angles: Non-standard and standard benchmarks in IoT systems.

1) Non-standard benchmarks in IoT systems:

- Standard Heuristics: Simple rule-based detection technique. For example, a notification on any mprotect call modifying a page to $W^{X}$, or an execve call originating from a dynamically apportioned memory region. Rule-based systems are a fast, simple, and effective method for known threats. They use predefined rules or signatures to identify threats. They can be powerful at detecting disclosed or simple attacks. However, their static nature makes them ill-suited for the dynamic landscape of IoT threats. They cannot detect zero-day attacks, which are crucial for IoT security. The rapid proliferation of new attack methods and IoT devices makes the rule’s set of updates impractical.
- Syscall N-gram Analysis: Conventional N-gram frequency analysis is used to detect anomalous sequences using a one-class SVM for outlier discovery Syscall n-gram SVM is an ML approach that models the behavior of an application program by observing sequences of system calls (syscalls). An SVM classifier is trained to differentiate between “normal” and “suspicious” syscall series. This technique is effective at detecting deviations from a program’s typical behavior, which can further indicate a shellcode injection.
- Adapted Enterprise Solutions: Due to resource limitations, a stripped-down version of an open-source EDR component was selected on higher-end IoT devices for a rudimentary comparison, (e.g., open-source OSQuery for process/memory snapshots, and the higher-end Raspberry Pi 4.)

Since the rule-based heuristics and syscall n-gram SVM are not considered standard benchmarks for IoT shellcode detection, we should also compare the framework with other standard methods used in IoT networks. While the “rule-based, n-gram SVM” is a part of the broader history of IDS and is used in research, they have significant limitations in the context of modern IoT.

2) Standard benchmarks in IoT systems: This type of benchmark needs to be replicable, provable, and widely accepted by the research community. While both methods have been explored in research, especially in early work, the current trend is toward more adaptable and practical models, such as those based on deep learning. In the context of IoT network security, CNNs, Long Short-Term Memory Networks (LSTMs), and datasets like UNSW-NB15 and CIC-IDS2017 are core components of modern, deep-learning-based IDS. They represent a shift away from conventional, signature-based techniques toward more intelligent, behavioral analysis. They can be described as follows:

- Convolutional Neural Networks: These CNNs are a type of deep learning model initially designed for video and image analysis. They excel at discovering spatial structures (patterns) and features. In the context of IoT network security, a CNN treats network traffic data (for example, a packet’s header and payload) as if it were a 1D image. It uses convolutional filters to automatically extract significant features, such as protocol anomalies and specific byte structures, that indicate suspicious behavior.
- Long Short-Term Memory Networks: This method is a specialized type of Recurrent Neural Network (RNN) designed to capture temporal dependencies and handle sequential data. They are handy for analyzing network traffic. An LSTM can remember past traffic patterns and use that memory to detect deviations that might trigger an attack. For example, it can learn from the regular sequential events in a device’s communication and trigger an alert when an unusual sequence of system calls occurs. Oftentimes, CNNs and LSTMs are merged in a hybrid model. While CNN extracts features from individual data points (e.g., a network packet), the LSTM observes the sequence of these features to identify multi-faceted attacks that unfold over time. The following well-known datasets are used to train and evaluate IDS. They were designed to address the shortcomings of existing datasets, which were often obsolete and did not accurately reflect real-world network traffic.
  – UNSW-NB15: This dataset was initiated in the Cyber Range Lab of the Australian Centre for Cyber Security. It consists of a mix of real, typical network activities and synthetically generated modern attacks, including Shellcode, Backdoors, Fuzzers, and Worms. It offers detailed network flow characteristics and is broadly used for research on IDS.
  – CIC-IDS2017: This component was initially developed by the Canadian Institute for Cybersecurity. It is then renowned for its realism, containing a week’s worth of network traffic with legitimate activities and contemporary known attacks, such as DoS, DDoS, Brute Force, and web application attacks. It includes several features, such as full packet payloads and flow characteristics extraction, making it an inclusive resource for evaluating IDSs.

The use of these datasets is essential for the growth of IoT security. They offer a standard benchmark for researchers to analyze, test, and compare the performance of different models, ensuring that new security measures are not only efficient but also generalizable to real-world threats.

### 4.5. Illustrative Graphs and Results

Key experimental results demonstrating the performance of our proposal framework.

**Figure 2:**
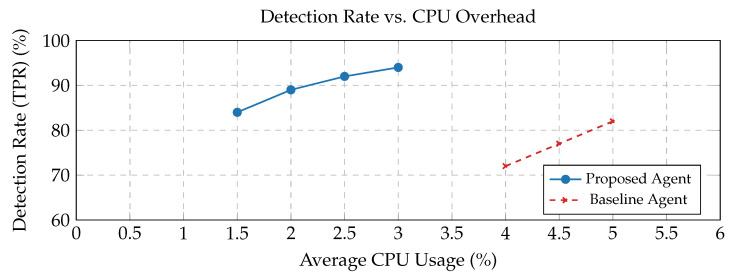
Detection rate vs. CPU overhead.

Figure 2 Detection Rate vs. CPU Overhead: This detection ratio illustrates the trade-off between the overall detection rate (TPR, Section 4.3) and the on-device agent’s CPU usage of the framework. Our proposed lightweight agent regularly achieves a high detection ratio with substantially lower CPU overhead compared to a hypothetical heavier baseline agent, demonstrating its appropriateness for IoT devices.

Table 1 displays the average CPU usage, deviation, and detection rate.

**Figure 3:**
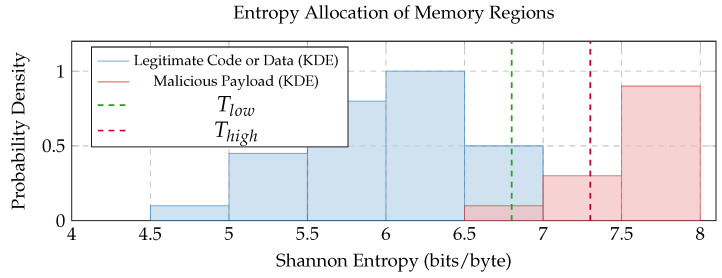
Entropy distribution.

The graph (Figure 3) represents the evaluation of the Shannon Entropy allocation.

Figure 3 Entropy Distribution of Memory Regions: This entropy allocation “Kernel Density Estimate (KDE)” visualizes the partitioning of Shannon Entropy values for memory regions. It highlights two distinct peaks: one at lower entropy values, corresponding to legitimate data, code, or scripts, and another at higher entropy values, delineating injected malicious shellcode. This distribution validates the effectiveness of Entropy-based Anomaly Scoring (EAS) as a key factor in anomaly detection. The dashed colored lines (green and red) indicate ordinary thresholds ($T_{low}$, $T_{high}$) used for allocation.

**Figure 4:**
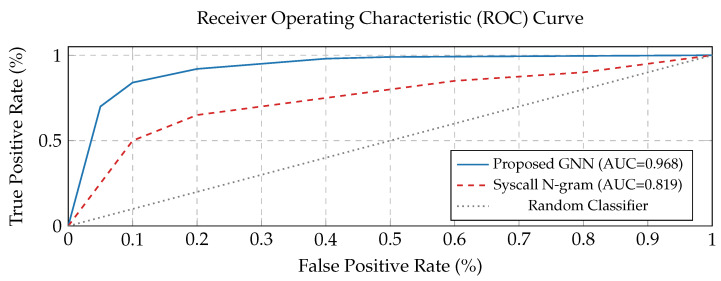
ROC curve.

Figure 4 Receiver Operating Characteristic (ROC) Curve: This scheme demonstrates the TPR against the FPR at distinct threshold settings for various detection techniques. The curve for our “Proposed GNN” (System Call Semantic Graph Analysis with GNN) is substantially closer to the top-left corner (higher AUC, with a value of 0.968), indicating greater performance in distinguishing between legitimate and malicious activities compared to the other two (Syscall N-gram and the random classifier).

**Figure 5:**
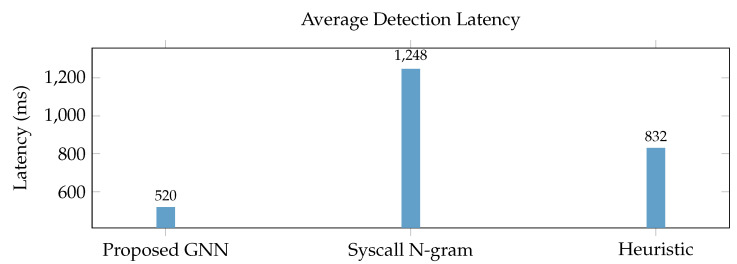
Average detection latency.

Figure 5 Average Detection Latency: This graph compares the average time taken from the execution of the in-memory shellcode to the time that the alert is triggered at the cloud backend for different techniques. Our “Proposed GNN” framework displays lower detection latency compared to the “Syscall N-gram” and “Heuristic” area under the curve (AUC) metrics due to adequate data aggregation at the edge and rapid processing by the GNN model, enabling faster response times. For more information on those concepts, please refer to [28,29,30,31,32].

**Figure 6:**
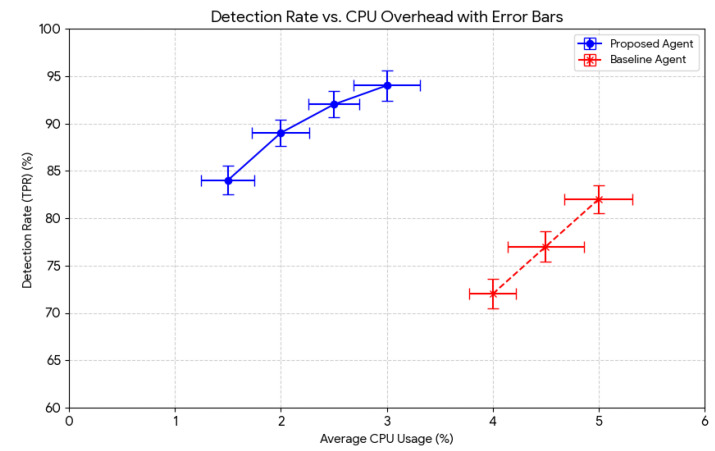
Error bars, calculated from the variance data in Table 1.

From the data in Table 1, the following values in Figure 6 are evaluated as follows:

## 5. Novelty and Innovations

Our research introduces several compelling novelties and innovations for in-memory shellcode detection in resource-constrained IoT environments:

1. IoT-Centric Lightweight Agent Model: Unlike other heavy endpoint detection and responses, our agent is purpose-built for extreme resource constraints. It primarily focuses on high-indicative factors, such as $W^{X}$ page detection, critical syscall monitoring, and adaptive entropy scoring with minimal calculation, ensuring device behavior (i.e., its functionalities) remains unimpeded. This selective telemetry gathering is also crucial for power-sensitive and CPU-limited devices.
2. Adaptive Entropy-based Anomaly Scoring (EAS): We moved beyond static entropy thresholds by proposing the use of device-specific baseline entropy profiles. By dynamically adapting $T_{low}$ and $T_{high}$ (e.g., using μ±kσ), we significantly decrease false positives while keeping up high sensitivity to anomalous code, which is critical for continuous monitoring in various IoT networks.
3. Hybrid Edge-Fog-Cloud Architecture Optimization: The proposed three-tiered architecture (i.e., “Edge” for lightweight data aggregation, “Fog” for optional aggregation/pre-processing, and “Cloud” for deep semantic analysis) is accurately optimized to allocate computational load effectively. This scheme leverages the strengths of each layer, expanding detection scalability and capability while diminishing the impact on the most limited devices. Unlike some other works [33,34], which primarily focused on edge-based ML for detecting botnets in IoT environments, and on intrusion detection systems, our approach deals with in-memory attacks, i.e., attacks that were built specifically to circumvent security solutions (AV, IDS, EDR, etc.). Please see [35] for more information.
4. System Call Semantic Graph Analysis with GNNs: This is an important innovation, as we transformed sequential system call events, raw, into semantic graphs that capture not only the temporal arrangement but also the causal dependencies and the contextual connections between calls and affected resources (i.e., memory regions, files, processes). The use of a customized GNN to evaluate these semantic graphs enables the detection of robust shellcodes (i.e., shellcodes designed to circumvent security measures) with a minimal false positive rate.

## 6. Discussion and Future Work

In-memory shellcode attacks are widely used in offensive security to evade hardened systems. Likewise, it can be used against IoT networks. Our proposal demonstrates a promising avenue for reinforcing and bolstering IoT security against this type of stealthy attack. The multi-layered technique, delivering computational load from constrained devices to advanced fog/cloud layers, is crucial for scalability and practicality.

However, several gaps necessitate future research and development:

- Hardware-Assisted Features Integration: A thorough enumeration of integration with incipient hardware security features is necessary to improve integrity, agent resilience, and probably offload some monitoring tasks.
- Adversarial ML Robustness: We will deepen our study on GNN-based detection against adversarial attacks built to perturb benign system calls, aiming to evade detection. We will also consider the nature of this type of detection-based feature, as those products usually lack heuristic capability.
- Automated Feature Engineering for GNNs: We will expand our research to extract optimal features for the GNN from various IoT device telemetry, adapting to new attack structures and device architectures.
- Device Attestation and Secure Boot Integration: We will dive into the concept of tying in-memory shellcode detection with secure boot processes and remote attestation means to ensure the integrity and authenticity of the monitoring agent and the original firmware.
- Decentralized Inference at Scale: Sometimes, in this type of IoT network, there may be some ultra-low latency requirements with periodic cloud connectivity. Thus, we will need to explore techniques that, if addressed, will help push more advanced GNN inference down to higher-end IoT gateways and AI accelerators.
- Dynamic Baseline Adaptation: Developing more robust techniques for both on-device and cloud layers to dynamically adapt baselines to modifying firmware updates, device workloads, and environmental shifts. That will also help reduce false-positive alerts and manual maintenance.

The “Hardware-Assisted Features Integration, Adversarial ML Robustness” and the “Automatic Feature Engineering for GNNs” are the three near-term commitments.

## 7. Conclusions

The increasing threat of in-memory shellcode runners poses a significant challenge to the security of IoT networks. This paper presents a comprehensive and novel approach that leverages a lightweight on-device agent for telemetry aggregation, and a cloud-based semantic analysis engine utilizing Graph Neural Networks to identify these stealthy attacks efficiently. By considering the evaluation of EAS for memory regions and SCSGA, we overcome the inherent constraints of IoT devices and conventional detection techniques. The framework employs a multi-layered approach to analyze various data points, which serve as the features for detection. The feature analytics of the framework are summarized as follows:

(a) “Entropy-based anomaly scoring”: this evaluation involves analyzing the randomness or predictability of memory regions. (b) “Lightweight behavioral monitoring”: we focused on observing the actions of processes. (c) “System Call Semantic Graph Analysis”: a novel method using Graph Neural Networks to analyze “system call patterns (e.g., Process ID, Hooking, Syscall ID, Win32 application programming interface).” (d) “Network behavior”: in the analysis phase, we also considered network activity to identify indicators of compromise.

Our innovations reduced false positives and enhanced detection accuracy while keeping minimal operational overhead. This research paper represents a significant step towards establishing a more secure and robust foundation for the future of interconnected IoT networks.

## Figures and Tables

**Table 1:** Standard deviation values exported from our excel sheet.

Agent	Average CPU USAGE (%)	Standard Deviation (CPU)	Average Detection Rate (%)	Standard Deviation Detection
Proposed agent	1.5	0.25	84	1.5
	2	0.27	89	1.42
	2.5	0.24	92	1.39
	3	0.31	94	1.59
Baseline agent	4	0.22	72	1.55
	4.5	0.36	77	1.61
	5	0.32	82	1.48

## Data Availability

The original contributions presented in this study are included in the article. Further inquiries can be directed to the corresponding authors.

## Abbreviations

The following abbreviations are used in this manuscript:

IoT	Internet of Things
GNN	Graph Neural Network
SCSG	System Call Semantic Graph Analysis
API	Application Programming Interface
AV	Antivirus
EDR	Endpoint Detection and Responses
CI	Command Injection
CPU	Central Processing Unit
RAM	Random Access Memory
AI	Artificial intelligence
DL	Deep Learning
ML	Machine Learning
CNN	Convolutional Neural Networks
SVM	Support Vector Machines
NLP	Natural Language Processing
TF-IDF	Term Frequency-Inverse Document Frequency matrices
BOW	Bag of Words
DLL	Dynamic-Link Library
JIT	Just-In-Time
IDS	Intrusion Detection System
NIDS	Network-based Intrusion Detection Systems
MQTT	Message Queuing Telemetry Transport
CoAPP	Constrained Application Protocol
HIDS	Host-based intrusion detection systems
OTA	Over-The-Air
WO, RO, EO	Writable, Readable, Executable-Only
CFI	Control Flow Integrity
SCSGA	System Call Semantic Graph Analysis
IPC	Inter-process Communication
GAT	Graph Attention Net-333 work
GCN	Graph Convolutional Network
C2	Command and Control
VLAN	Virtual Local Area Network
NASM	Netwide Assembler
DDoS	Distribution Denial of Services
SSH	Secure Shell
HTTP	Hypertext Transfer Protocol
RSS	Resident Set Size
KDE	Kernel Density Estimate
AUC	Area Under the Curve
